# Lactate level during cardiopulmonary bypass as a predictor of postoperative outcomes in adult patients undergoing cardiac surgery

**DOI:** 10.1186/s40981-016-0064-3

**Published:** 2016-11-24

**Authors:** Satoko Noguchi, Junichi Saito, Eiji Hashiba, Tetsuya Kushikata, Kazuyoshi Hirota

**Affiliations:** Department of Anesthesiology, Hirosaki University Graduate School of Medicine, 5 Zaifucho, Hirosaki, 036-8562 Japan

**Keywords:** Blood lactate level, Cardiac surgery, ICU-free survival days

## Abstract

**Background:**

It has been reported that prolonged intensive care unit (ICU) stay after cardiac surgery is associated with poor patient outcome. In addition, prolonged stay can block the efficient use of ICU beds with an increase in expenditure of health-care costs. The aim of the present study was to retrospectively determine which pre- and intra-operative factors could significantly affect ICU-free survival days (IFSD) which has been suggested to reflect postoperative patients’ outcome, as well as variables significantly associated with the main predictors of IFSD.

**Findings:**

We reviewed anesthesia charts and medical records of 145 patients undergoing cardiac surgery under cardiopulmonary bypass (CPB) in our hospital from January 2014 to October 2015, and 72 patients’ records were finally used for the analysis. IFSD was a median of 25 days (95% CI 24–26). The multiple regression analysis indicated that preoperative estimated glomerular filtration rate, differences between preoperative mean arterial pressure and mean CPB pressure, and blood lactate level at 2 h after CPB (CPB-2 h) were independently associated with IFSD (β regression coefficients 0.086, −0.083, and −3.601, respectively).

**Conclusion:**

In addition to preoperative renal function and differences between preoperative MAP and CPB pressure, the lactate level at CPB-2 h could be a major predictor of postoperative outcome in patients undergoing cardiac surgery.

## Findings

### Background

It has been reported that prolonged intensive care unit (ICU) stay after cardiac surgery is associated with poor patient outcome [1, 2]. As prolonged ICU stay has also been reported to cause a significant decrease in long-term survival, determination of risk factors related to the prolonged ICU stay may be helpful to improve patients’ outcome by avoiding these risk factors. In addition, prolonged stay can block the efficient use of ICU beds, may result in postponement of other operations, and also increases expenditure of health-care costs [1, 2]. The use of life-support equipments such as ventilator and renal replacement system can particularly cause excessive expenditure in the ICU [3].

When patients die in a short period in the ICU, length of ICU stay (LOS) becomes shorter. Thus, as the LOS may not reflect patient’s prognosis and severity of an illness, “ICU-free survival days” is now widely used as a better index than LOS for the outcome study [4, 5].

The aim of the present study was to retrospectively determine which pre- and intra-operative factors could significantly affect ICU-free survival days.

### Materials and methods

#### Study subjects

This is a retrospective observational study approved by the Medical Ethics Committee of Hirosaki University Graduate School of Medicine (approval number 2015-168). There were 145 patients undergoing elective cardiac surgery under CPB in our institution between January 2014 and October 2015. As the following patients were excluded: patients under 18 years old, receiving on-pump beating procedure or circulatory arrest, and with CPB duration less than 120 min, 72 patients’ records were finally used for the analysis.

#### Anesthesia and CPB management

Anesthesia was induced and maintained using total intravenous anesthesia with propofol 1–5 mg/kg/h, ketamine 0.5–2 mg/kg/h, fentanyl 15–25 μg/kg, and intermittent 10 mg rocuronium in all cases. After tracheal intubation, the lungs were ventilated with intermittent positive-pressure ventilation under F_I_O_2_ of 0.3 to 1 which was dependent on oxygenation status. Vasoactive agents and inotropics were administrated to maintain stable hemodynamics when needed. The whole-blood activated coagulation time (ACT) was maintained greater than 400 s with intermittent intravenous heparin administration during CPB. The CPB circuit was primed with colloid solutions at variable volumes. Cold crystalloid cardioplegia was administrated in an antegrade fashion every 30 min during the cross-clamping period or whenever electrical activity resumed. During CPB, the following conditions were maintained: a mild hypothermia of 32–34 °C, nonpulsatile flow at 2.2–2.6 L/min/m^2^, mean perfusion pressure of 60–70 mmHg, and hematocrit (Hct) >22%. To keep target mean perfusion pressure during CPB, vasopressor administration and/or increasing perfusion flow were performed. At the end of CPB, protamine was administrated as required to return the ACT to the baseline values. Intra-operative blood salvage was also performed in most cases.

#### Data collection and definitions

The following available data were collected from each patient’s record: age, sex, body weight, height, comorbidity (hypertension, diabetes, kidney injury, cerebrovascular diseases), current smoking and history of cardiac surgery, preoperative estimated glomerular filtration rate (eGFR), preoperative serum brain natriuretic peptide, New York Heart Association class, left ventricular ejection fraction (%) estimated by echo-cardiography or angiography, type of operation, CPB data (CPB duration, aortic cross-clamping duration, nadir Hct, nadir temperature, perfusion pressure, mean flow), blood transfusions, preoperative vasoactive medication, LOS, and postoperative morbidity and mortality. Postoperative morbidity was defined as the following: renal complications (more than “injury” of RIFLE criteria for acute kidney injury (AKI)); respiratory complications (mechanical ventilation longer than 24 h with no apparent cardiac reason, requirement of noninvasive positive pressure ventilation or high-flow nasal cannula after tracheal extubation, acute respiratory distress syndrome); neurologic complications (coma or new focal neurologic deficit) [6]; cardiovascular complications (cardiac arrest, the requirement of percutaneous cardiopulmonary support (PCPS)); and requirement of surgical hemostasis. Blood lactate was measured by an arterial blood gas analyzer (ABL 800, Radiometer Co., Copenhagen, Denmark). After CPB was started, blood lactate was measured every 1 h. Average mean arterial pressure (MAP) was obtained from three preoperative blood pressure readings: on admission, in the day before surgery, and just before induction of general anesthesia was defined as baseline MAP [7]. Mean CPB pressure and flow were also defined as the average of MAP and flow reading at every 30 min during CPB. Ratio of <CPB 60 or 50 mmHg was defined as the rate of the duration lower than 60 or 50 mmHg during CPB. ICU-free survival days was determined as the reminder to subtract LOS from 28 days. The criteria of ICU stay for post-cardiac surgical patients were the following: unstable respiration without any respiratory assist, using vasopressors, or needing renal replacement therapy. The discharge criteria of ICU were the following: stable respiration without orotracheal tube and maintaining SpO_2_ >92% without noninvasive positive pressure, stable circulation with no requirement of hemodynamic monitoring and maintaining systolic blood pressure >90 mmHg without vasoactive agents, and no requirement of compensation of acute abnormal laboratory values.

#### Outcome and statistical analyses

To determine which pre- and intra-operative factors could affect the ICU-free survival days, we performed univariate analysis to find candidate factors (*p* < 0.05) and then did multivariate regression analysis (SPSS 24.0, SPSS Inc., Chicago, IL, USA) by stepwise method. Data are presented as regression coefficient with 95% confidence intervals (CI). Other data were shown as median [25% quartile–75% quartile] or *n* (%). A *p* < 0.05 was considered statistically significant.

### Results

Preoperative and intra-operative demographics are depicted in Tables 1 and 2, respectively. ICU-free survival days in our study was a median of 25 days (95% CI 24–26). There were 19 patients (26.4%) who had at least one postoperative complications, 3 patients with renal complications, 12 patients with respiratory complications, 2 patients with neurologic complications, 3 patients with cardiovascular complications, and 4 patients required surgical hemostasis. And there were two patients (2.8%) who had died in ICU.

**Table 1:** Preoperative patient demographics

Variable	Data
Age (years)	66.5 [60.5–73.0]
Weight (kg)	53.2 [48.0–60.3]
Body mass index (kg/m^2^)	22.3 [20.2–23.6]
Body surface area (m^2^)	1.52 [1.43–1.63]
Gender: male (%)	34 (47.2)
Hypertension (%)	49 (68.0)
Diabetes (%)	12 (16.7)
Dyslipidemia (%)	26 (36.1)
Current smoking (%)	29 (40.3)
Previous stroke (%)	18 (25.0)
Chronic dialysis (%)	9 (12.5)
Previous cardiac surgery (%)	8 (11.1)
Preoperative eGFR (ml/min/1.73 m^2^)	54.1 [44.1–69.1]
Preoperative BNP (pg/ml)	253 [87–496]
NYHA class I/II/III/IV	16/45/10/1
Left ventricular ejection fraction (%)	61.5 [50.7–70.6]
Preoperative systolic blood pressure (mmHg)	131 [116–141]
Preoperative diastolic blood pressure (mmHg)	68 [60–76]
Preoperative mean arterial pressure (mmHg)	87 [82–97]
Preoperative β-blocker (%)	19 (26.4)
Preoperative anticoagulant (%)	29 (40.3)
Preoperative antiplatelet drug (%)	12 (16.7)
Type of operation	
Single valve surgery (%)	43 (59.7)
Complex valve surgery (%)	16 (22.2)
CABG and valve surgery (%)	9 (12.5)
Other (%)	4 (5.6)

Data were shown as median [25% quartile–75% quartile] or *n* (%)

*eGFR* estimated glomerular filtration rate, *BNP* brain natriuretic peptide, *NYHA* New York Heart Association

**Table 2:** Intra-operative characteristics

Variable	Data
CPB duration (min)	198 [167–232]
Aortic cross-clamping duration (min)	133 [112–161]
Nadir hematocrit on CPB (%)	24.1 [20.5–27.8]
Nadir temperature on CPB (°C)	34.0 [33.0–34.4]
Mean CPB pressure (mmHg)	55.0 [49.7–60.5]
Ratio of <CPB pressure 60 mmHg (%)	65.7 [40.0–89.9]
Ratio of <CPB pressure 50 mmHg (%)	26.0 [9.2–47.6]
Preoperative MAP—mean CPB pressure	32.0 [12.7–51.3]
Mean CPB flow (L/min)	3.77 [3.48–4.13]
Mean CPB flow index (L/min/m^2^)	2.49 [2.41–2.55]
Vasopressors during CPB (%)	23 (31.9)
Transfusions during CPB with RBCs (%)	26 (36.1)
Transfusions during CPB with FFPs (%)	20 (27.8)
Lactate level at CPB-0 h (mmol/L)	0.8 [0.6–1.0]
Lactate level at CPB-1 h (mmol/L)	1.1 [0.8–1.4]
Lactate level at CPB-2 h (mmol/L)	1.3 [1.0–1.7]

Data were median [25% quartile–75% quartile] or *n* (%), ratio of <CPB pressure **mmHg (%): (duration of lower than **mmHg during CPB/total CPB time) × 100

*CPB* cardiopulmonary bypass, *MAP* mean arterial pressure, *CPB-#h* # h after CPB started

The univariate analysis indicated that ICU-free survival days could significantly be associated with preoperative eGFR, CPB duration, differences between preoperative MAP and mean CPB pressure, and blood lactate level measured 2 h after CPB started (CPB-2 h) (Table 3).

**Table 3:** Univariate association of preoperative and intra-operative variables with the ICU-free survival days

Variable	Regression coefficient [95% CI]	*p* value
Gender: male	−0.397 [−2.608 to 1.741]	0.692
Age	−0.028 [−0.191 to 0.055]	0.813
Body mass index	−0.216 [−0.426 to 0.0169]	0.069
Hypertension	0.738 [−1.463 to 3.181]	0.463
Diabetes	−0.114 [−3.083 to 2.749]	0.910
Dyslipidemia	−0.059 [−2.330 to 2.196]	0.953
Previous stroke	0.649 [−1.688 to 3.318]	0.518
Current smoking	−1.411 [−3.734 to 0.639]	0.163
Previous cardiac surgery	−0.415 [−4.173 to 2.736]	0.679
Chronic dialysis	1.940 [−1.279 to 14.99]	0.088
Preoperative eGFR	0.074 [0.036 to 0.111]	<0.001*
Preoperative BNP	−0.068 [−0.469 to 0.046]	0.568
Left ventricular ejection fraction	0.177 [−0.120 to 0.494]	0.137
Preoperative anticoagulant	−0.719 [−3.004 to 1.413]	0.475
Valve surgery	0.620 [−3.262 to 6.203]	0.537
CPB duration	−0.260 [−0.538 to −0.147]	0.028*
Aortic cross-clamping duration	−0.109 [−0.419 to 0.067]	0.361
Nadir hematocrit	0.179 [−0.061 to 0.321]	0.133
Mean CPB pressure	0.042 [−0.211 to 0.201]	0.727
Ratio of <CPB pressure 60 mmHg	−0.184 [−0.350 to 0.144]	0.123
Ratio of <CPB pressure 50 mmHg	−0.057 [−0.269 to 0.174]	0.636
Preoperative MAP—mean CPB pressure	−0.235 [−0.427 t6 0.172]	0.047*
Mean CPB flow index	−0.190 [−0.455 to 0.115]	0.110
Vasopressors during CPB	−0.134 [−2.487 to 2.175]	0.894
Transfusions during CPB with RBCs	1.800 [−0.332 to 5.195]	0.082
Lactate level at CPB-0 h	−0.094 [−0.298 to 0.070]	0.431
Lactate level at CPB-1 h	−2.120 [−4.566 to 0.327]	0.088
Lactate level at CPB-2 h	−2.646 [−4.455 to −0.837]	0.005*

Data were shown as regression coefficients with 95% confidence intervals (CIs), ratio of <CPB pressure **mmHg (%): (duration of lower than **mmHg during CPB/total CPB time) × 100

eGFR estimated glomerular filtration rate, *BNP* brain natriuretic peptide, *CPB* cardiopulmonary bypass, *MAP* mean arterial pressure, *CPB-#h* # h after CPB started

*p < 0.05

In our multivariate linear regression model including possible confounders, preoperative eGFR, differences between preoperative MAP and mean CPB pressure, and blood lactate level at 2 h after CPB were independently associated with ICU-free survival days (Table 4).

**Table 4:** Multivariate association of preoperative and intra-operative variables with the ICU-free survival days

Variable	Regression coefficient	*p* value
Preoperative eGFR	0.086	<0.001*
CPB duration	−0.107	0.278
Preoperative MAP—mean CPB pressure	−0.083	0.015*
Lactate level at CPB-2 h	−3.601	<0.001*

Model *R* ^2^ = 0.387; data were shown as β regression coefficients

*CPB* cardiopulmonary bypass, *MAP* mean arterial pressure, *CPB-#h* # h after CPB started, *eGFR* estimated glomerular filtration rate

*p < 0.05

### Discussion

Our retrospective analysis suggests that preoperative eGFR, differences between preoperative MAP and mean CPB pressure, and blood lactate level at CPB-2 h were associated with ICU-free survival days in the multivariate analysis.

The increase in blood lactate during CPB is well known in patients undergoing cardiac surgery due to variable reasons such as peripheral circulatory failure, hemodilution, or using catecholamine [8–10]. In addition, high blood lactate levels during perioperative period have been reported to be associated with worse outcomes including mortality [9–17]. Previous studies showed that early postoperative hyperlactatemia was more sensitive predictor of mortality and morbidity for patient undergoing cardiac surgery under CPB than late hyperlactatemia during ICU stay [10, 18]. It was also reported that the maximum lactate level during cardiac surgery after CPB was significantly associated with LOS [9] and ICU-free survival days [19]. Early postoperative hyperlactatemia was associated with a low oxygen delivery during CPB [9–12]. Thus, the proper managements of CPB could avoid the increase in blood lactate levels. Although previous studies showed that the highest lactate level during CPB, its level at the end of CPB, or its level at the postoperative period could be related to worse postoperative outcomes, there is no report showing that lactate level at some time point during CPB could be the predictor of postoperative impaired outcome. In this regard, the present study may be the first report. In addition, the present data suggest that early therapeutic intervention in accordance with blood lactate levels during CPB might be important to avoid worth postoperative outcome rather than therapeutic intervention after CPB or during ICU stay.

In the present study, difference between preoperative MAP and mean CPB perfusion pressure was also significantly associated with ICU-free survival days. A previous study suggested that a drop in MAP by more than 26 mmHg from preoperative blood pressure caused a 2.8-fold increase in a risk of development of postoperative AKI [7]. It is likely that the patients with development of AKI stay in the ICU longer than those without AKI. The maintenance of adequate perfusion pressure during CPB is important for vital organ perfusion [19]. It has been suggested that relatively high perfusion pressure during CPB is preferable to prevent cerebrovascular and gastroenterological complications, especially in the patients with impaired autoregulation system due to arteriosclerosis [13]. Even if autoregulation system well regulates to maintain peripheral tissue circulation, CPB perfusion pressure seems to be more important to prevent peripheral tissue hypoperfusion under nonpulsatile flow. Recently, study suggested that not only high perfusion flow but also high perfusion pressure was necessary during CPB to protect the skeletal muscle [20]. It remains controversial which is more important for management of CPB, pressure, or flow. However, the present study clearly demonstrates that it may be necessary to minimize the difference preoperative MAP and CPB perfusion pressure. Further study will be required to determine the cutoff point between them for postoperative morbidity.

Preoperative renal function also independently contributes to ICU-free survival days. A previous study suggested that preoperative serum creatinine was a predictor for postoperative renal complications and in-hospital mortality in patient undergoing cardiac surgery [6, 21]. In addition, it has also suggested that CPB duration would prolong in patients with preoperative renal insufficiency [6, 21]. As patients requiring hemodialysis particularly have multiple comorbidities such as hypertension, angina pectoris, diabetes, hyperlipidemia, and cerebrovascular diseases with severe arteriosclerosis, these patients would need prolongation of CPB and extended ICU stay. Therefore, we also have to pay careful attention to preoperative renal function in cardiac surgical patients.

Mean ICU-free survival days in present study was 25 days in our institution. Furushima and colleagues [14] reported that maximum lactate levels measured after CPB was independently associated with ICU-free survival days. And other study also suggested that postoperative maximum lactate levels could be the predictors about cardiac surgical morbidity and mortality. Demers and colleagues [9] showed that the patient with peak intra-operative blood lactate levels higher than 4.0 mmol/L needed prolonged ICU stay longer than 5 days. Maillet and colleagues [10] suggested that blood lactate level of 3 mmol/L at ICU admission could indicate high risk of postoperative morbidity and mortality in cardiac surgical patients. In addition, they showed that the LOS was 3.8 days in early hyperlactatemia group and 1.9 days in normal lactate range group. Compared to ICU-free survival days calculated from the LOS in the previous reports, the present data were slightly different. This small discrepancy might be due to the difference in the ICU discharge criteria between each institution. Other pre- or intra-operative predictors for cardiac surgical morbidity were reported as follows: emergency case, re-operation, preoperative low cardiac output, and prolonged CPB duration [2, 6]. In the present study, although emergency cases were excluded for the analysis, re-operation (*p* = 0.679 by univariate analysis), preoperative low cardiac output (*p* = 0.137 by univariate analysis), and CPB duration (*p* = 0.028 by univariate analysis but *p* = 0.278 by multivariate regression analysis) were not recognized as significant. Therefore, although CPB duration might become significant with much larger sample size, others would not be predictors for postoperative morbidity and mortality in cardiac surgical patients.

The sample size of this study was small, but we performed post hoc analysis and the power of this study was 0.88 (*n* = 72, *α* = 0.05, *r* = −0.31, correlated regression between ICU-free survival days and lactate level measured 2 h after CPB). Therefore, we believe that the present data must be meaningful.

In conclusion, in addition to preoperative renal function and differences between preoperative MAP and CPB pressure, the lactate level at CPB-2 h could be a predictor of ICU-free survival days in patients undergoing cardiac surgery.
